# X-ray Scintillation in Lead Halide Perovskite Crystals

**DOI:** 10.1038/srep37254

**Published:** 2016-11-16

**Authors:** M. D. Birowosuto, D. Cortecchia, W. Drozdowski, K. Brylew, W. Lachmanski, A. Bruno, C. Soci

**Affiliations:** 1 CINTRA UMI CNRS/NTU/THALES 3288, Research Techno Plaza, 50 Nanyang Drive, Border X Block, Level 6, 637553 Singapore; 2 Center of Disruptive Photonic Technologies, TPI, SPMS, Nanyang Technological University, 21 Nanyang Link, 637371 Singapore; 3 Interdisciplinary Graduate School, Nanyang Technological University, 639798 Singapore; 4 Energy Research Institute @ NTU (ERI@N), Research Techno Plaza, Nanyang Technological University, 50 Nanyang Drive, 637553 Singapore; 5 Institute of Physics, Faculty of Physics, Astronomy and Informatics, Nicolaus Copernicus University, Grudziadzka 5, 87-100 Torun, Poland; 6 School of Physical and Mathematical Sciences, Division of Physics and Applied Physics, Nanyang Technological University, 21 Nanyang Link, 637371 Singapore

## Abstract

Current technologies for X-ray detection rely on scintillation from expensive inorganic crystals grown at high-temperature, which so far has hindered the development of large-area scintillator arrays. Thanks to the presence of heavy atoms, solution-grown hybrid lead halide perovskite single crystals exhibit short X-ray absorption length and excellent detection efficiency. Here we compare X-ray scintillator characteristics of three-dimensional (3D) MAPbI_3_ and MAPbBr_3_ and two-dimensional (2D) (EDBE)PbCl_4_ hybrid perovskite crystals. X-ray excited thermoluminescence measurements indicate the absence of deep traps and a very small density of shallow trap states, which lessens after-glow effects. All perovskite single crystals exhibit high X-ray excited luminescence yields of >120,000 photons/MeV at low temperature. Although thermal quenching is significant at room temperature, the large exciton binding energy of 2D (EDBE)PbCl_4_ significantly reduces thermal effects compared to 3D perovskites, and moderate light yield of 9,000 photons/MeV can be achieved even at room temperature. This highlights the potential of 2D metal halide perovskites for large-area and low-cost scintillator devices for medical, security and scientific applications.

The investigation of X-ray detectors started with the discovery of X-rays by Wilhelm Röntgen, who noticed the glow from a barium platino-cyanide screen placed besides a vacuum tube^1^,^2^. Since this discovery, more than one hundred years ago, the development of efficient^3^,^4^,^5^ and large-area^5^,^6^,^7^ X-ray detectors has been a topic of continuous interest, targeting a wide range of applications, from crystallography^8^ to space exploration^9^.

Modern X-ray detectors rely on two main mechanisms of energy conversion. The first is photon-to-current conversion, in which a semiconducting material directly converts the incoming radiation into electrical current^4^,^5^,^6^; the second is X-ray to UV-visible photon down-conversion, in which a scintillator material is coupled to a sensitive photodetector operating at lower photon energies^2^. Both methods are equally compelling for practical implementations, although their viability will ultimately depend on the development of new materials to overcome some of the current limitations, such as high cost, small area, and low conversion efficiency of the X-ray absorbers. Recent demonstrations of the use of hybrid metal-halide perovskites for X- and γ-ray detection has spurred great interest in this class of materials^7^,^10^,^11^,^12^. Besides their good detection efficiency, solution processing holds great promise for facile integration and development of industrial and biomedical applications.

Methylammonium lead trihalide perovskites (MAPbX_3_ where MA = CH_3_NH_3_ and X = I, Br, or Cl) have demonstrated excellent performance in optoelectronic devices like field effect transistors^13^, highly sensitive photodetectors for visible region^14^, and light emitting devices^15^,^16^. Moreover, compositional tuning was used to realize tunable-wavelength lasers^17^. As X-ray detectors, MAPbX_3_ yield notably large X-ray absorption cross section due to large atomic numbers of the heavy Pb and I, Br, Cl atoms^10^,^11^. Thin-film MAPbX_3_ p-i-n photodiode and lateral photoconductor devices have shown good efficiency for X-ray photon-to-current conversion^10^,^11^. However, thin-film X-ray detectors have typically low responsivity at high (keV) photon energies, where the absorption length (~mm) is much larger than the film thickness (~*μ*m); even if thickness is increased to improve detection probability, direct photon-to-current conversion is ultimately hampered by the limited carrier-diffusion length (~1 *μ*m in perovskites)^10^. Efficient X-ray photon-to-current conversion has been shown recently in single-crystal (thick) perovskite MAPbBr_3,_ but sensitivity is still limited to energies up to 50 keV^11^. Also, standard γ-photon counting for energies up to 662 keV has been demonstrated in MAPbI_3_^12^.

As opposed to direct photon-to-current conversion detectors, X-ray scintillators do not suffer from limited carrier diffusion length of the absorbing material^18^,^19^. Thin films of phenethylammonium lead bromide, PhE-PbBr_4_, with sub-nanosecond scintillation decay time have been previously tested in X-ray^20^ and proton^21^ scintillators, but yielded only 5–6% detection efficiency of 60 keV X-rays, limited by the film thickness (200 *μ*m)^21^. By combining the good high-energy response with large absorption cross section deriving from large thickness and high mass-density, single crystal perovskite scintillators are therefore expected to improve detection efficiency of keV X- or γ-rays.

In this paper, we present a thorough comparative study of the scintillation properties of three-dimensional (3D) and two-dimensional (2D) low-bandgap perovskite single crystals. We have synthesized mm-scale 3D perovskite crystals MAPbI_3_ and MAPbBr_3_, and 2D perovskite crystal (EDBE)PbCl_4_ (EDBE = 2,2^′^-(ethylenedioxy)bis(ethylammonium)), comprising of alternating organic and inorganic layers which form a multi-quantum-well-like structure. The excellent quality of these crystals is indicated by structural analysis and by the very small density of shallow traps (*n*_*0*_ ~ 10^5^–10^7^ cm^−3^, *E* ~ 10–90 meV) determined by X-ray excited thermoluminescence, which reduces after-glow effects. Thanks to their lower bandgap compared to traditional scintillator crystals^6^, perovskite crystals produce extremely high light yields of >120,000 photons/MeV (as estimated from X-ray-excited luminescence) at low temperature. In 3D perovskites, the light yield is greatly reduced at room temperature (<1,000 photons/MeV) due to strong thermal quenching effects. Conversely, the 2D perovskite crystal is far more robust against thermal quenching thanks to its large exciton binding energy (~360 meV) induced by charge confinement within the inorganic layers. These results confirm the excellent properties of metal-halide perovskites for X-ray detection, and highlight the potential of 2D perovskite crystals with large exciton binding energy for high-light yield X-ray scintillators.

## Results and Discussion

**Figure 1:**
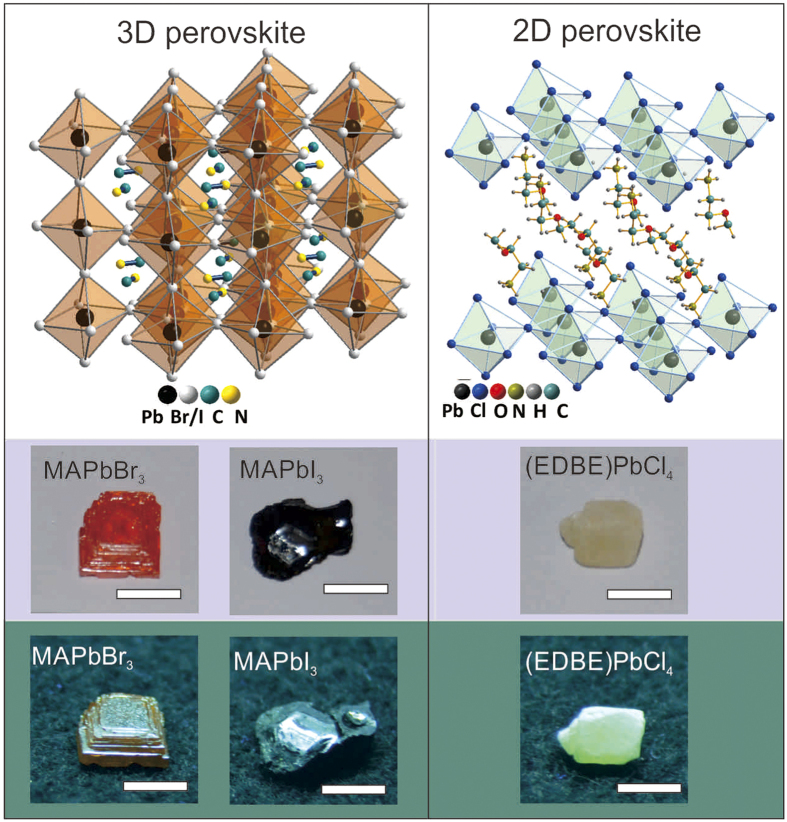
Crystal structure and appearance. Top row: crystal structure representation of MAPbX_3_ (X = I, Br) three-dimensional perovskites (left), and (EDBE)PbCl_4_ two-dimensional perovskite (right); Middle row: photographs of the large single crystals of hybrid lead halide perovskites; Bottom row: glow of the crystals under ultraviolet lamp excitation. Scale bars: 5 mm.

To study scintillation performance, we have synthesized the high-quality, large-size (~30 to 100 mm^3^) perovskite single crystals shown in Fig. 1 (see *Materials and methods* section for details on crystal growth). MAPbX_3_ (X = I, Br) crystals have the conventional three dimensional ABX_3_ perovskite structure, consisting of a continuous network of corner sharing PbX_6_^4−^ octahedra with methyl-ammonium cations occupying the interstitial sites^22^,^23^. XRD patterns of the ground crystals confirm the formation of the desired perovskites MAPbBr_3_ and MAPbI_3_, having cubic and tetragonal crystal structure, respectively (see Supplementary Figs S1 and S2). Conversely, (EDBE)PbCl_4_ belongs to the general class of APbX_4_ (X = I, Br, Cl and A = bidentate organic cation) “two-dimensional” perovskite crystals^24^; it consists of the stack of <100> -oriented perovskite inorganic layers forming a 2D Pb-X network in alternation with organic sheets of di-ammonium cations EDBE^2+^ (Fig. 1). The presence of pronounced 001 and higher order 00 l reflections in the XRD pattern indicates unequivocally the formation of the layered perovskite with monoclinic crystal structure (see Supplementary Fig. S3). To the naked eye MAPbI_3_, MAPbBr_3_, and (EDBE)PbCl_4_ crystals appear lustrous black, orange, and white, respectively. The corresponding glows under ultraviolet lamp excitation are green and white for MAPbBr_3_ and (EDBE)PbCl_4_ crystals, while the glow of MAPbI_3_ could not be observed since its emission lies in the near infrared. Crystal colors and glows agree well with the absorption and photoluminescence properties of the corresponding thin films, which show optical energy gaps of *E*_g_ = 1.51, 2.18, and 3.45 eV for MAPbI_3_^22^, MAPbBr_3_ and (EDBE)PbCl_4_^24^, respectively (Supplementary Fig. S4).

Perovskite crystals offer multiple advantages for X-ray scintillation, specifically: i. Since the light yield of X-ray scintillation is inversely proportional to the optical bandgap *E*_g_^2^,^18^, low-bandgap perovskites of MAPbI_3_, MAPbBr_3_ and (EDBE)PbCl_4_ are expected to yield up to about 270,000, 190,000, and 120,000 photons/MeV, respectively. Those light yields are much higher than state-of-art cerium (Ce^3+^) doped lanthanum tribromides LaBr_3_ (*E*_g_ = 5.90 eV)^25^,^26^ and Ce^3+^ doped lutetium iodides LuI_3_ (*E*_g_ = 4.15 eV)^27^,^28^ scintillators, with light yields of 68,000 and 100,000 photons/MeV, respectively. ii. Since X-ray absorption length scales with the effective atomic number *Z*_eff_ and mass density *ρ*^2^, MAPbI_3_, MAPbBr_3_, and (EDBE)PbCl_4_ (*Z*_eff_ = 66.83, 67.13, and 67.52, *ρ* = 3.947, 3.582, and 2.191 gr⁄cm^3^, respectively) should reach X-ray absorption lengths up to 1 cm at 1 MeV, similar to Ce^3+^-doped LaBr_3_ and LuI_3_ scintillators (see Supplementary Fig. S5). iii. The unusually large Stokes shift of two-dimensional (EDBE)PbCl_4_ could be particularly beneficial to the scintillation yield^29^, which is substantially reduced by self-absorption of the luminescence^30^. iv. The extremely fast photoluminescence decay of MAPbI_3_, MAPbBr_3_, and (EDBE)PbCl_4_ (fast components of 4.3, 0.8–5.2, and 7.9 ns, respectively) may provide faster scintillation than 15 ns of Ce^3+^-doped LaBr_3_^25^,^30^ and 33 ns of Ce^3+^-doped LuI_3_^28^ (Supplementary Fig. S6). Nanosecond scintillation decay times were indeed demonstrated in PhE-PbBr_4_ using X-ray and γ-ray pulses, consistent with time-resolved photoluminescence^20^,^31^. v. Finally, long emission wavelengths in the range of 400 to 700 nm allow optimal detection of scintillation using highly sensitive avalanche photodiodes (APDs), which can reach quantum efficiencies up to 90–100% in comparison with photomultipliers (PMTs) with only 40–50% efficiency^28^.

**Figure 2:**
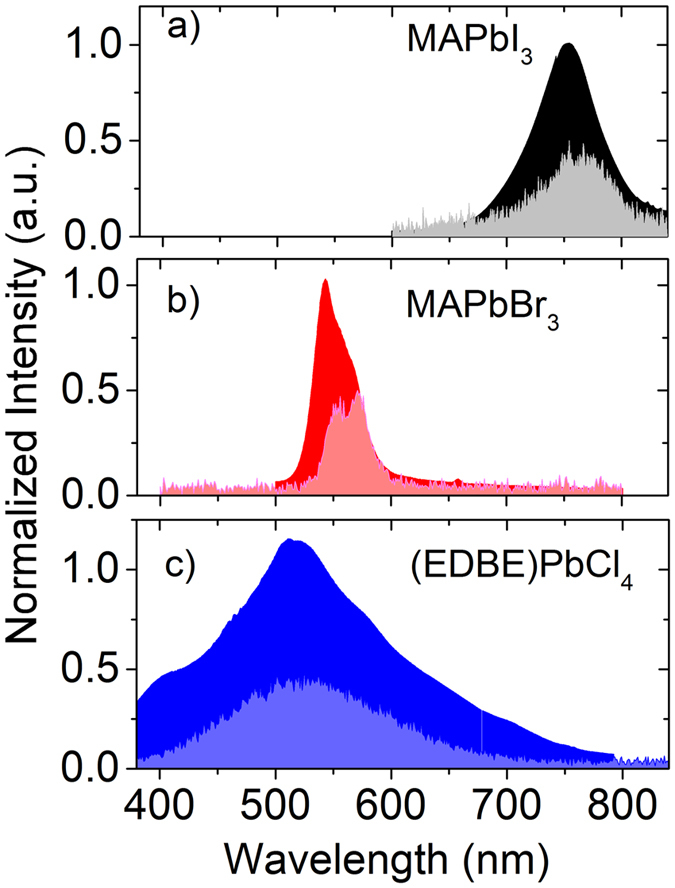
Emission spectra under X-ray and optical excitation. X-ray excited luminescence (light color area) and photoluminescence (dark color area) spectra of (**a**) MAPbI_3_, (**b**) MAPbBr_3_, and (**c**) (EDBE)PbCl_4_ recorded at room temperature with excitation wavelengths for photoluminescence of 425, 500, and 330 nm, respectively. Photoluminescence and X-ray excited luminescence spectra were normalized to their maxima, and normalized X-ray excited luminescence spectra were divided by a factor of two for clarity.

The X-ray excited luminescence and photoluminescence spectra of MAPbI_3_, MAPbBr_3_, and (EDBE)PbCl_4_ crystals recorded at room temperature are shown in Fig. 2 (see experimental details in *Materials and methods* section). Both X-ray excited luminescence and photoluminescence spectra of MAPbI_3_ have a single broadband peak centered at 750 nm with FWHM of ~80 nm (Fig. 2a). For MAPbBr_3_, both X-ray excited luminescence and photoluminescence spectra exhibit double peaks centered around 560 and 550 nm, respectively. MAPbBr_3_ has the narrowest emission band with full width of half-maximum (FWHM) of ~40 nm (Fig. 2b). On the other hand, (EDBE)PbCl_4_ has the broadest emission band centered at 520 nm, with FWHM of ~160 nm (Fig. 2c). Based on emission wavelength, MAPbBr_3_ and (EDBE)PbCl_4_ appear to be the most promising candidates for the scintillators coupled to APD^28^.

In all perovskite crystals, X-ray excited luminescence and photoluminescence spectra are very similar, indicating that the dominant scintillation mechanism is straightforward: upon X-ray absorption, high-energy excitations thermalize through ionizations and excitations of atoms, until excitons are generated at energies near the bandgap. X-ray excited luminescence stems solely from the intrinsic excitonic emission of the perovskites, and no other defect states seem to be involved in the scintillation process.

The dynamics of radiative processes in materials under high-energy excitation is often complicated by slower non-exponential components due to charge carrier trapping and re-trapping, which manifest themselves as delayed luminescence, or afterglow. Upon termination of the X-ray excitation, afterglow effects would typically contribute a residual luminescence background with characteristic lifetime of few ms, thus lowering the effective light yield and worsening the signal-to-noise ratio. Afterglow effects are particularly detrimental for applications like computed tomography, in which temporal crosstalk considerably reduces the image quality^2^. Charge carrier trapping and re-trapping processes can be monitored by thermoluminescence measurements. In our specific mode of operation for thermoluminescence intensity measurements, we were able to record steady-state X-ray excited luminescence intensity during irradiation, immediately prior to the thermoluminescence scan (see details in *Materials and methods*). In this way, two distinct integrated intensities can be evaluated: the first one, which we denote as *I*_*TL*_, comprising the range from the end of X-ray irradiation till the end of the entire run, while the second one, denoted as *I*_*TL*_ + *I*_*ssXL*_, comprising the range from the start of the X-ray irradiation until the end of the run. This allows calculating, for each sample, the *I*_*TL*_/(*I*_*TL*_ + *I*_*ssXL*_) ratio, which can be interpreted as the fraction of the total excitation energy accumulated into traps^19^,^32^. The value of this ratio, therefore, provides a qualitative estimate of the influence of traps on the scintillation yield.

**Figure 3:**
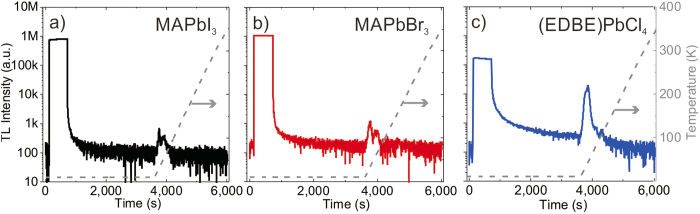
Residual luminescence background after X-ray excitation. Low temperature thermoluminescence cur ves of (**a**) MAPbI_3_, (**b**) MAPbBr_3_, and (**c**) (EDBE)PbCl_4_. The data are presented on a time scale starting at temperature of 10 K and increasing to 350 K after 3600 s, as indicated by the dashed line in the right panel (temperature scale on the right axes).

Typical thermoluminescence curves of the metal halide perovskite crystals are shown as solid curves in Fig. 3. After termination of the X-ray excitation at 10 K, long tails extending to thousands of seconds were observed in all crystals. Although the long-lived component of this afterglow effect is much slower than the photoluminescence decay (see Supplementary Fig. S6), it only occurs at low temperatures (~10 K) and is negligible at room temperature. In the case of MAPbI_3_ and MAPbBr_3_, low temperature thermoluminescence curves are dominated by a double-structured peak, with two smaller satellite peaks appearing at longer times (Fig. 3a and b). In (EDBE)PbCl_4_, the low-temperature thermoluminescence curve shows that one peak strongly dominates the other peak while the total intensity of the peaks is much higher than those in MAPbI_3_ and MAPbBr_3_ (Fig. 3c). The ratio of *I*_*TL*_/(*I*_*TL*_ + *I*_*ssXL*_) ~ 0.002 is very similar in both MAPbI_3_ and MAPbBr_3_, which is extremely low in comparison with other oxide materials used for scintillators, such as lanthanide aluminium perovskite or garnets^19^,^32^,^33^,^34^. Moreover, MAPbI_3_ and MAPbBr_3_ crystals show nearly trap-free behavior from T = 75 K up to the highest temperature investigated of T = 350 K, a very desirable characteristic from the point of view of scintillation speed and efficiency. In (EDBE)PbCl_4_, *I*_*TL*_/(*I*_*TL*_ + *I*_*ssXL*_) ~ 0.058, a slightly higher value than in the three-dimensional perovskite crystals, but still relatively low.

**Figure 4:**
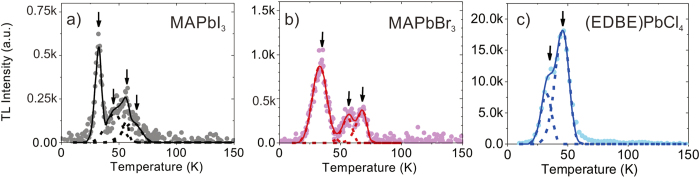
Determination of low-energy trap states. Glow curves of (**a**) MAPbI_3_, (**b**) MAPbBr_3_, and (**c**) (EDBE)PbCl_4_ recorded after 10 min X-ray irradiation at 10 K, at a heating rate of 0.14 K/s. The solid lines are the best fits to the experimental data points by multiple Randall-Wilkins equations (Eq. 1): single components and peak temperatures (*T*_max_) are indicated by dashed lines and arrows, respectively (see Table 1 for fitting parameters).

The zero-order glow curves of the three crystals are presented in Fig. 4. Appearance of thermoluminescence signal at temperatures below 150 K reveals the existence of low-energy trap states. Since for such states it is difficult to determine the exact number of traps, their depth and frequency factors^33^, we restrict our analysis to thermoluminescence peaks with intensity larger than 10% of the maximum. Thermoluminescence curves have been deconvoluted into *k* glow peaks, based on the classic Randall-Wilkins equation^35^:


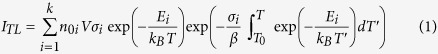


where *T* is the temperature, *β* the heating rate, and *k*_*B*_ the Boltzmann constant; *n*_0*i*_ is the initial trap concentration, *V* is the crystal volume, *E*_*i*_ the trap depth, *σ*_*i*_ the frequency factor of each component. Note that the unitless number of traps *n*_0*i*_*V* is often used to compare the afterglow of different crystals^19^,^32^,^33^,^34^,^35^.

This analysis provides a good indication of the characteristics of prevailing trap states, however it cannot resolve the existence of traps that fall at times much longer than seconds, or with mixed order kinetics^32^. The room temperature lifetime of trapped carriers, such as electron or hole centers and excitons, *τ*_*i*_, can also be estimated from the energy and frequency factor of the trap, using the well-known Arrhenius formula:


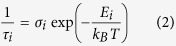


**Figure 5:**
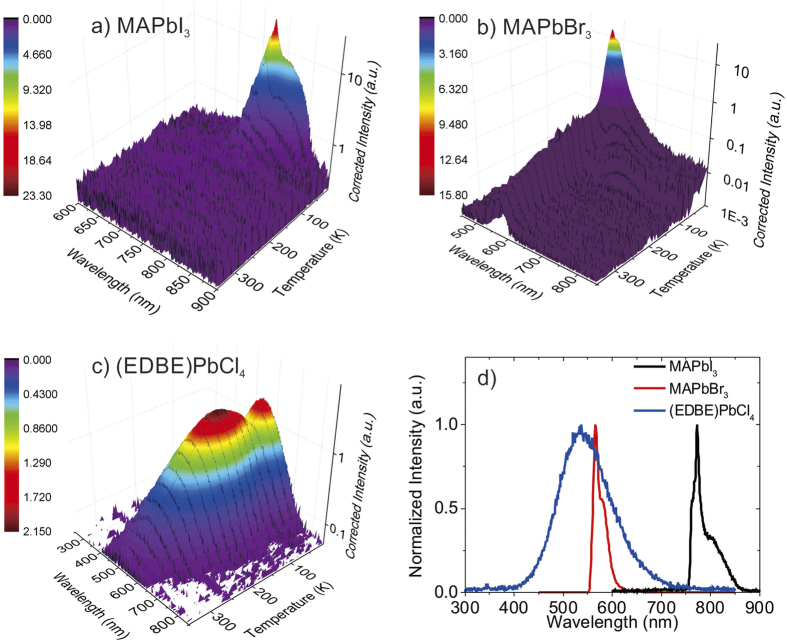
Temperature dependent luminescence under X-ray excitation. X-ray excited luminescence spectra (X-ray excited luminescence) of perovskite crystals at various temperatures, from 10 to 350 K: (**a**) MAPbI_3_, (**b**) MAPbBr_3_, and (**c**) (EDBE)PbCl_4_. (**d**) Comparison of the normalized X-ray excited luminescence spectra at T = 10 K.

While the glow curves of MAPbI_3_ and MAPbBr_3_ in Fig. 4a and b have been fitted using four and three components, respectively, the glow curve of (EDBE)PbCL_4_ in Fig. 4c could be fitted with only two components. The corresponding fitting parameters are shown in Table 1. All crystals have relatively low trap densities, with depth energy (*E*) varying from ~10 to 90 meV. The initial trap concentrations *n*_0_ in MAPbI_3_ and MAPbBr_3_ can be calculated from the total number of traps (*n*_0_*V*~10^3^–10^4^) and the volume of the crystal (*V*~30–100 mm^3^). The resulting trap concentrations (*n*_0_~10^5^–10^7^ cm^−3^) are comparable to those of shallow traps previously observed in photoconductivity measurements (~10^5^–10^7^ cm^−3^)^11^ and space-charge-limited-current (~10^9^–10^10^ cm^−3^)^23^, also considering the uncertainty in the estimate of the active crystal volume. The fastest room temperature lifetimes (*τ*) of MAPbI_3_ and MAPbBr_3_ are of the order of milliseconds, long enough to contribute to the light yield components without residual luminescence background. Correspondingly, logarithmic frequency factors (ln *σ*) are all below 16, which is much smaller than ln *σ*~30 typically found in pristine or activated oxide materials^19^,^32^,^33^,^34^, also reported in Table 1 for comparison. (EDBE)PbCl_4_ has the largest trap density among the perovskites we investigated, *n*_0_ ~ 10^7^ cm^−3^. Large concentration of shallow traps may be beneficial for X-ray scintillation at low-temperature, as in the case of Ce^3+^-doped YAlO_3_ and LuAlO_3_^34^, or pristine Li_2_B_4_O_7_^36^. This is indeed seen in temperature dependent X-ray excited luminescence spectral maps shown in Fig. 5.

MAPbI_3_ (Fig. 5a) and MAPbBr_3_ (Fig. 5b) show strong dependence of X-ray excited luminescence on temperature, with significantly reduced emission at temperatures above 100 K. At very low temperatures they display distinct emission bands with sharp maxima at 770 nm and 540 nm, respectively (see Fig. 5d for comparison of X-ray excited luminescence spectra recorded at T = 10 K). Notably, the X-ray excited luminescence peak at 770 nm, with FWHM of 5 nm, has the same characteristics of coherent light emission previously observed in MAPbI_3_^17^. Side bands also appear at 760 and 800 nm in MAPbI_3_ and at ~590 nm in MAPbBr_3_, but their origin is still unclear. The X-ray excited luminescence spectrum of (EDBE)PbCl_4_ (Fig. 5c) consists of a much broader band peaking at ~520 nm, with intensity significantly less sensitive to temperature. As temperature increases, the X-ray excited luminescence intensity first decreases between 10 and 50 K, then increases towards 130 K, and reduces steadily at higher temperatures. In all crystals, the FWHM of X-ray excited luminescence peaks increases with increasing temperature, consistent with the spreading of excited electrons to high vibrational levels^37^.

As discussed previously, the light yield of perovskite single crystals estimated from their bandgaps should be >120,000 photons/MeV. From the pulse height spectra of samples excited with 662 keV *γ*-ray of Cs^137^ shown in Supplementary Fig. S7, the actual light yield of (EDBE)PbCl_4_ at room temperature is moderate, with ~9,000 photons/MeV. We note that there are not so many reports about the energy spectra from *γ*-ray reported for perovskite scintillator^10^ and direct conversion detector^12^. Light yield of (EDBE)PbCl_4_ is actually similar to that of two-dimensional perovskite crystal PhE-PbBr_4_ (10,000 photons/MeV) reported previously^31^. The light yields of MAPbBr_3_ and MAPbI_3_ at room temperature are much lower, and cannot be extracted from pulse height experiments. Low light yields at room temperature may arise from thermally activated quenching effects. To confirm this hypothesis, we have derived light yields at different temperatures from the integrated intensities of the X-ray excited emission spectra in Fig. 5; considering the small afterglow, we expect the light yield derived from X-ray excited emission spectra to be very similar to that derived from pulse height spectra.

**Figure 6:**
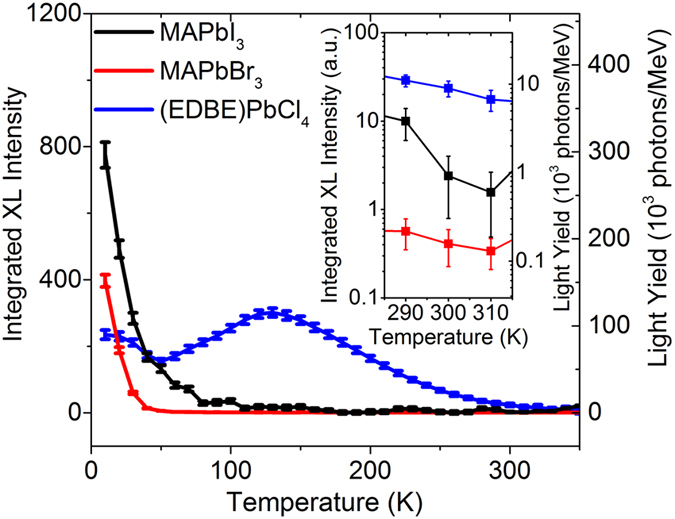
Temperature dependence of the light yields. Light yields of MAPbI_3_, MAPbBr_3_, and (EDBE)PbCl_4_ obtained from the integrated X-ray excited luminescence intensities at various temperatures, from 10–350 K. The left axis shows integrated intensity in arbitrary units obtained from the corrected X-ray excited luminescence spectra in Fig. 5, while the right axis exhibits the light yield in absolute units after calibration of the light yield of (EDBE)PbCl_4_ at 300 K to ~9,000 photons/MeV, as derived independently from its pulse height spectrum. The inset shows details of the curves from 290 to 310 K.

Light yields derived from the integrated X-ray excited luminescence emission intensities of the three halide perovskite crystals as a function of temperature are reported in Fig. 6. We integrated the corrected intensity of X-ray excited luminescence spectra in Fig. 5 and used the light yield of ~9,000 photons/MeV derived from the pulse height spectrum of (EDBE)PbCl_4_ at 300 K (Supplementary Fig. S7) to calibrate the integrated intensity. For (EDBE)PbCl_4_, the resulting light yield at 300 K is about ~8% of the maximum at 130 K. Since the light yield is linearly proportional to the photoluminescence quantum efficiency^18^ while the efficiency of charge transport to the recombination center is almost unity^24^,^29^, the ratio of 8% is consistent with reported (EDBE)PbCl_4_ photoluminescence quantum efficiency of less than 10% at room temperature. Light yields of MAPbI_3_ and MAPbBr_3_ are <1,000 photons/MeV at room temperature (see inset of Fig. 6) and the light yields at 10 K are 296,000 and 152,000 photons/MeV, respectively. The maximum light yields of MAPbI_3_ and MAPbBr_3_ correspond well to the attainable light yields derived from bandgaps of 270,000 and 190,000 photons/MeV, respectively. Unlike (EDBE)PbCl_4_, the ratio between the light yields at 300 K and those at 10 K of less than 0.5% for MAPbI_3_ and MAPbBr_3_ are much smaller than their respective quantum efficiencies of 30% and 10%^17^,^38^. Additional light-yield-loss in MAPbI_3_ and MAPbBr_3_ could be due to non radiative recombination of free electrons and holes within the ionization tracks^2^,^18^.

The larger light yield of (EDBE)PbCl_4_ at room temperature can be explained by its extremely large exction-binding energy of about 360 meV^29^, which is typical of 2D perovskites and gives rise to a pronounced excitonic absorption below the band-edge (see Supplementary Fig. S4). In contrast, 3D perovskites are known for their low exciton binding energy - for MAPbBr_3_ and MAPbI_3_ in the range of 2–70 meV^22^,^23^. Loosely bound excitonic states in 3D perovskites are much more prone to thermal quenching than tightly bound excitons in 2D perovskites like (EDBE)PbCl_4_, implying that 3D perovskite crystals require much lower operating temperatures than 2D crystals to achieve optimal scintillation performance.

## Conclusions

Our findings confirm that hybrid lead halide perovskite single crystals are very promising scintillator materials in terms of low fabrication costs, low intrinsic trap density, nanosecond fast response, and potentially high light yield. Thermoluminescence measurements have indicated that perovskite crystals have much lower trap density than conventional oxide scintillator materials^19^,^33^. Low-temperature X-ray excited luminescence measurements have shown that the X-ray luminescence yield can be as high as ~120,000 photons/MeV in (EDBE)PbCl_4_ at T = 130 K, and in excess of 150,000 photons/MeV in MAPbI_3_ and MAPbBr_3_ at T = 10 K. The wide synthetic versatility of hybrid perovskites allows easy tuning of their scintillation properties: for example, their emission spectra can be controlled by cation or halide substitution to perfectly match the spectral sensitivity of high-quantum-efficiency APD, like in the case of MAPbBr_3_ and (EDBE)PbCl_4_. Their emissive properties can be further enhanced through engineering of perovskite structure and dimensionality: while light yield of 3D perovskites MAPbI_3_ and MAPbBr_3_ is significantly reduced at room temperature (<1,000 photons/MeV), the 2D perovskite (EDBE)PbCl_4_ is less affected by thermal quenching (~9,000 photons/MeV at room temperature), thanks to its large exciton binding energy. Given the potential of hybrid lead halide perovskite crystals, further efforts should be made to synthesize new materials for X- and *γ*-ray scintillation: for instance, the light yield of perovskite crystals could be further improved by introduction of lanthanide ions, e.g. Ce^3+^ ions, as impurities^18^,^39^, or mixing the halides to modify the bandgap^40^, while the optimal operating temperature could be increased through the design of wide band gap 2D perovskite crystals with minimal quenching effects.

## Materials and Methods

### X-ray scintillation measurements

The main setup used for X-ray excited luminescence and thermoluminescence measurements consists of an Inel XRG3500 X-ray generator (Cu-anode tube, 45 kV/10 mA), an Acton Research Corporation SpectraPro-500i monochromator, a Hamamatsu R928 PMT, and an APD Cryogenics Inc. closed-cycle helium cooler. The emission spectra were corrected for the transmittance of the monochromator and the quantum efficiency of the PMT. First, we recorded X-ray excited luminescence at various temperatures, between 10 and 350 K. We note that the measurements were carried out from 350 K to 10 K to avoid possible contributions from thermal release of charge carriers to the emission yield. After X-ray excited luminescence measurements, we measured low temperature thermoluminescence and glow curves. Prior to thermoluminescence measurements, the samples were exposed to X-rays for 10 min at 10 K. Thermoluminescence and glow curves were recorded between 10 and 350 K at a heating rate of about 0.14 K/s. Thermoluminescence curves were recorded with the monochromator set to the zeroth order. Photoluminescence spectra were recorded with a commercial spectrofluorometer HORIBA Jobin Yvon Fluorolog-3 spectrofluorometer at room temperature.

### Crystal growth

Three-dimensional perovskite precursors, MABr and MAI, were synthetized by mixing hydrobromic acid (48% wt in water, Sigma-Aldrich) and hydroiodic acid (57% wt in water, Sigma-Aldrich) with methylamine solution (CH_3_NH_2_, 40% in methanol, Tokyo Chemical Industry, Co., Ltd) in 1:1 molar ratio. The ice-cooled mixture was left under magnetic stirring for 2 h, and the solvent removed with a rotary evaporator. The resulting powders were dissolved in ethanol, crystallized with diethylether for purification repeating the cycle 6 times, and finally dried in vacuum oven at 6 °C for 12 h. For (EDBE)PbCl_4_(EDBE = 2,2′-(ethylenedioxy)bis(ethylammonium)), the organic precursor (EDBE)Cl_2_ was synthetized in aqueous solution by reaction of 2,2′-(ethylenedioxy)bis(ethylamine) (98%, Sigma Aldrich) with excess of HCl (37% in H_2_O). The solution was stirred for 4 h at room temperature to complete the reaction. A purification process similar to that discussed for MABr and MAI was applied to collect the final white and high purity powders.

For the synthesis of hybrid perovskite crystals, the following inorganic precursors were purchased from Sigma-Aldrich: lead(II) chloride (PbCl_2_, 99.999%), lead(II) bromide (PbBr_2_, 99.999%) and lead(II) iodide (PbI_2_, 99.0%). Crystals of MAPbBr_3_ were synthetized using inverse temperature crystallization as similarly reported elsewhere^27^. 2 ml of 1 M DMF solution of MABr and PbBr_2_ (1:1 molar ratio) were left overnight on a hotplate (110 °C) without stirring, allowing the precipitation of the perovskite crystals. MAPbI_3_ were obtained by slow evaporation at room temperature of a saturate *N*,*N*-dimethylformamide (DMF) solution of MAI and PbI_2_ (1:1 molar ratio). To obtain (EDBE)PbCl_4_ crystals, a 1 M solution of (EDBE)Cl_2_ and PbCl_2_ (1:1 molar ratio) in dimethylsulphoxide (DMSO) was prepared by dissolving the precursors at 110 °C on a hotplate. After natural cooling of the solution at room temperature, slow crystallization over a period of 1 month results in the formation of cm-scale white perovskite crystals. The crystallization processes were performed under inert N_2_ atmosphere. All the obtained crystals were collected from the precursor solutions, washed with diethylether and dried in vacuum overnight.

## Additional Information

**How to cite this article**: Birowosuto, M. D. *et al.* X-ray Scintillation in Lead Halide Perovskite Crystals. *Sci. Rep.* **6**, 37254; doi: 10.1038/srep37254 (2016).

**Publisher’s note**: Springer Nature remains neutral with regard to jurisdictional claims in published maps and institutional affiliations.

## Supplementary Material

Supplementary Information

## Figures and Tables

**Table 1:** Trap state parameters.

Compound	*T*_max_ (K)	*E* (eV)	ln *σ* (s^−1^)	*τ* (s)	*n*_0_*V*	Reference
MAPbI_3_	32	0.0309	8.09	1.04⋅10^−3^	2.45⋅10^4^	This work
	46	0.0226	1.78	0.41	1.85⋅10^4^	
	56	0.0901	15.60	5.95⋅10^−4^	6.12⋅10^3^	
	62	0.0389	3.25	0.18	1.45⋅10^4^	
MAPbBr_3_	33	0.0139	1.16	0.54	7.61⋅10^4^	This work
	56	0.0602	9.02	1.31⋅10^−3^	2.10⋅10^4^	
	68	0.0909	12.1	2.04⋅10^−4^	2.73⋅10^4^	
EDBEPbCl_4_	32	0.0177	2.83	0.12	5.95⋅10^5^	This work
	45	0.0281	3.40	0.10	1.71⋅10^6^	
LuAlO_3_: Ce^3+^	36	0.0148	0.9346	2.16⋅10^−2^	2.84⋅10^4^	19, 32, 34
	88	0.105	10.07	2.29⋅10^−2^	1.53⋅10^4^	
	187	0.498	27.22	2.51⋅10^−2^	2.10⋅10^6^	
	206	0.385	17.56	1.61⋅10^−2^	4.64⋅10^4^	
	223	0.669	30.99	2.18⋅10^−2^	1.38⋅10^4^	
	253	0.75	30.53	2.08⋅10^−2^	4.84⋅10^4^	
	273	0.799	30.08	2.05⋅10^−2^	1.52⋅10^5^	
YAlO_3_: Ce^3+^	108	0.30	29.24	4.99⋅10^−2^	~10^5^	19, 32, 34
	154	0.50	34.18	3.02⋅10^−2^	~10^5^	
	281	0.421	18.05	1.95	~10^4^	
Gd_3_Al_2_Ga_3_O_12_: Ce^3+^	36	0.0576	15.9	1.2⋅10^−6^	1.6⋅10^5^	33
	45	0.0446	8.32	1.4⋅10^−3^	4.7⋅10^5^	
	73	0.116	15.1	2.7⋅10^−5^	3.4⋅10^5^	
	181	0.211	9.01	0.52	2.9⋅10^5^	
	240	0.527	21.31	0.65	2⋅10^5^	
	255	0.321	9.76	19	7.5⋅10^5^	

The parameters were derived from the fitting of first-order glow curves in Fig. 4: *T*_max_ is the temperature at which the glow curve peaks, *E* the trap depth, ln *σ* the logarithmic frequency factor in *s*^−1^, *τ* the room temperature lifetime, and *n*_*0*_*V* the total, initial number of traps. Comparative parameters of known scintillator materials from the literature are also reported in the last three lines.
