# Supplementary material for: X-ray Scintillation in Lead Halide Perovskite Crystals
Source: Sci Rep. 2016 Nov 16;6:37254. doi: 10.1038/srep37254 (PMC5111063; doi:10.1038/srep37254)
Supplement: Supplementary Information [file srep37254-s1.doc]

**Supplementary Information**

**X-ray Scintillation in Lead Halide Perovskite Crystals**

M. D. Birowosuto,^1,2,*^ D. Cortecchia,^3,4^ W. Drozdowski,^5^ K. Brylew,^5^ W. Lachmanski,^5^ A. Bruno,^4^ C. Soci^2,4,6,*^

*^1^CINTRA UMI CNRS/NTU/THALES 3288, Research Techno Plaza, 50 Nanyang Drive, Border X Block, Level 6, Singapore 637553*

*^2^Center of Disruptive Photonic Technologies, TPI, SPMS, Nanyang Technological University, 21 Nanyang Link, Singapore 637371*

*^3^Interdisciplinary Graduate School, Nanyang Technological University, Singapore 639798*

^4^ *Energy Research Institute @ NTU (ERI@N), Research Techno Plaza, Nanyang Technological University, 50 Nanyang Drive, Singapore 637553*

^5^*Institute of Physics, Faculty of Physics, Astronomy, and Informatics, Nicolaus Copernicus University, Grudziadzka 5, 87-100 Torun, Poland*

*^6^ School of Physical and Mathematical Sciences, Division of Physics and Applied Physics, Nanyang Technological University, 21 Nanyang Link, Singapore 637371*

**Corresponding authors:* [*mbirowosuto@ntu.edu.sg*](mailto:mbirowosuto@ntu.edu.sg)*;* [*csoci@ntu.edu.sg*](mailto:csoci@ntu.edu.sg)

### X-ray Diffraction

To characterize the crystal structure of the perovskite crystals, X-ray powder diffraction (XRPD) was performed on perovskite powders obtained from ground crystals on a BRUKER D8 ADVANCE with Bragg-Brentano geometry using Cu K*_α_* radiation (*λ* = 1.54,056 Å), step increment of 0.02^○^ and 1 s of acquisition time. The results are shown in Figs. S1, S2 and S3.

**Figure S1**. XRD pattern of MAPbBr_3_ powders. The diffractogram is consistent with the perovskite structure having cubic crystal system, space group *Pm*3*m* and lattice parameters a = 5.917(1) Å.


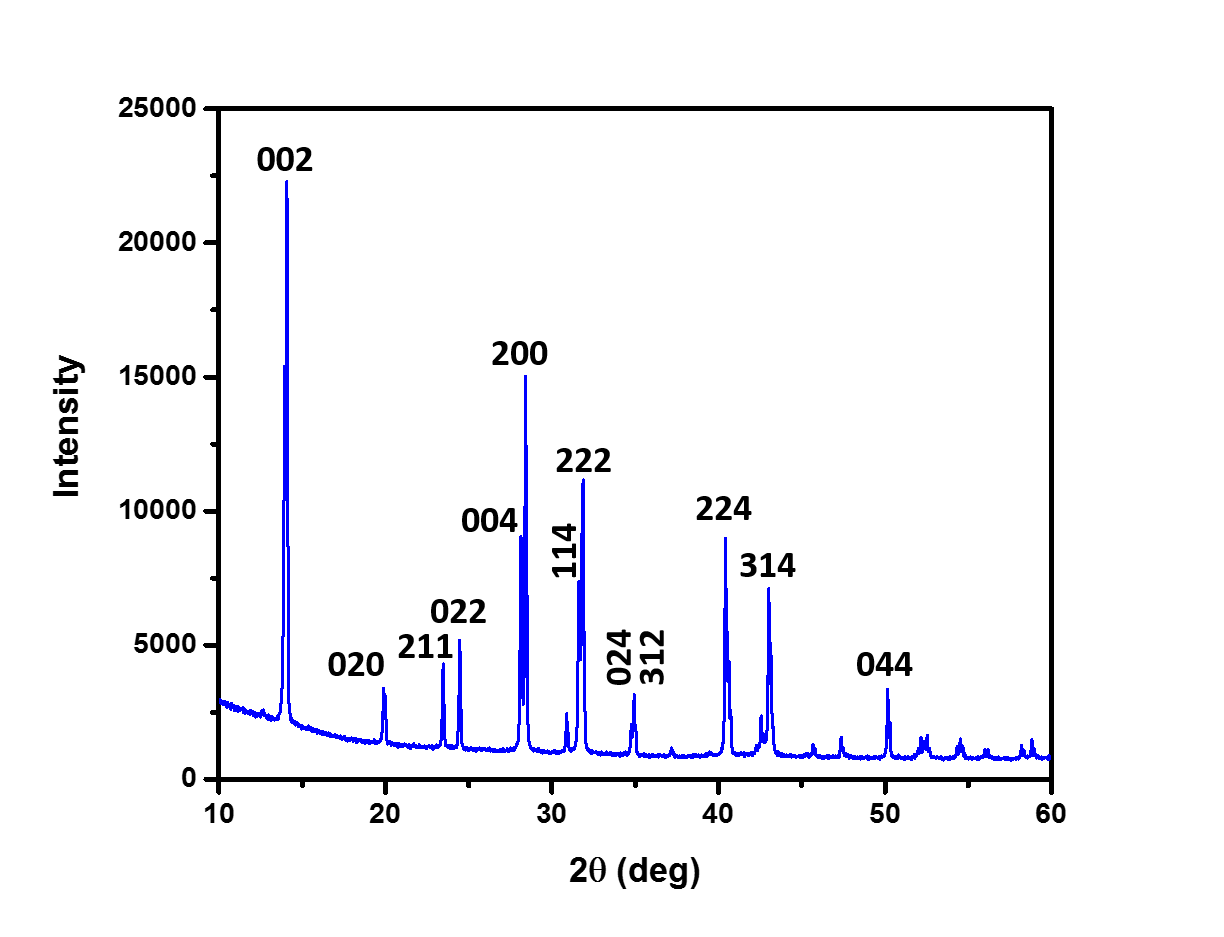


**Figure S2.** XRD pattern of MAPbI_3_ powders. The diffractogram is consistent with the perovskite structure having tetragonal crystal system, space group *I*4 ⁄ *mcm* and lattice constants a = 8.867(5) Å and b = 12.649(3) Å.

**Figure S3.** XRD pattern of (EDBE)PbCl_4_ powders. The 00l reflections indicate the formation of the layered structure of the two-dimensional perovskite, in agreement with the previously reported structure (by Dohner *et al,* [1] having monoclinic crystal system, space group C2 and refined lattice parameters a = 7.762(8) Å, b = 7.629(2) Å, c = 13.375(7) Å, *β* = 102.7(2) Å.

### Absorption and Photoluminescence

Absorption and photoluminescence (PL) measurements were performed in order to obtain the energy band gap and confirm the large Stoke shift in two-dimensional perovskite scintillators (Fig. S4). Absorption spectra were recorded by an UV-VIS-NIR spectrophotometer (UV3600, Shimadzu) using a scanning resolution of 0.5 nm. Steady-state photoluminescence spectra were recorded by a Fluorolog-3, (HORIBA Jobin Yvon) spectrofluorometer with wavelength resolution 0.5 nm.


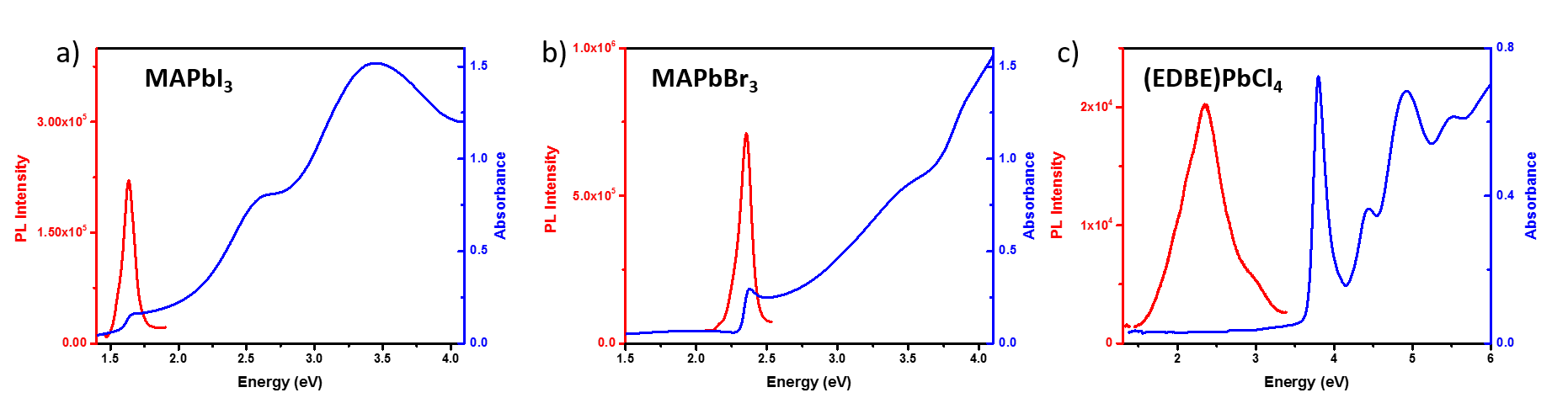


**Figure S4**. Room temperature absorption (blue) and photoluminescence (PL) spectrum (red) of a) MAPbI_3_, b) MAPbBr_3_, c) (EDBE)PbCl_4_ (thin films). The PL peak blue-shifts from 760 nm to 527 nm from MAPbI_3_ to MAPbBr_3_, following the corresponding blue-shift of the absorption edges. The absorption spectrum of (EDBE)PbCl_4_ shows a pronounced excitonic peak at 326 nm and broadband, highly Stoke-shifted PL peaked at 525 nm.

### Absorption Length

Three types of the interaction mechanisms for electromagnetic radiation in matter play an important role in the absorption of X- and γ-rays. These are photoelectric absorption, Compton scattering, and pair production. All these processes lead to the partial or complete absorption of the radiation quantum. The absorption length was obtained from formula [2]:

where *Ze_ff_, N_A_,* *ρ*, and *σ* are the effective atomic number, Avogadro number, the mass density, and the absorption cross section for each atomic element. As the cross section was separately characterized for photoelectric, Compton scattering, and pair production, the total absorption length was determined by the inverse sum of the absorption lengths for the three interaction mechanisms mentioned above (Fig. S5).


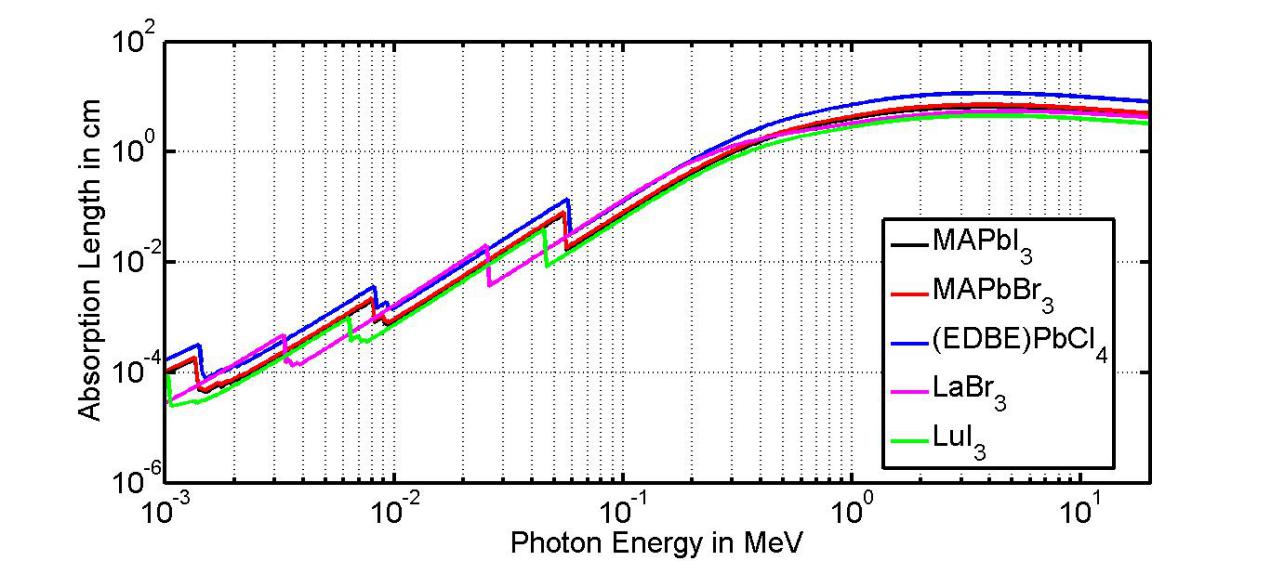


**Figure S5.**Calculated absorption length of perovskite crystals as a function of photon energy, covering the X-ray spectral region. Curves for Ce^3+^ doped LuI_3_ [2] and LaBr_3_ [3] scintillators are also added for comparisons.

### Time Resolved Photoluminescence

### The micro-PL setup is based on free space excitation technique with the excitation path and the emission collection from the side, using a VIS-NIR microscope objective (40x, NA=0.65). The MAPbI3, MAPbBr3, and (EDBE)PbCl4 single crystals were excited with 5-MHz-repetition-rate, picosecond-pulse light sources at 640 and 370 nm of Edinburgh laser diodes and at 330 nm of a Picoquant light-emitting diode, respectively. In all cases, the beam spot size was about 2 mm. A silicon-based charge-coupled-device camera was used for imaging. Time-resolved decay curves were obtained using grating Edinburgh Instruments or tunable bandpass filters at 766, 540, and 520 nm for MAPbI3, MAPbBr3 and (EDBE)PbCl4 crystals, respectively. The signal from the Hamamatsu photomultiplier or Micro Photon Devices single-photon avalance photodiode was acquired by a time-correlated single photon counting card.

###
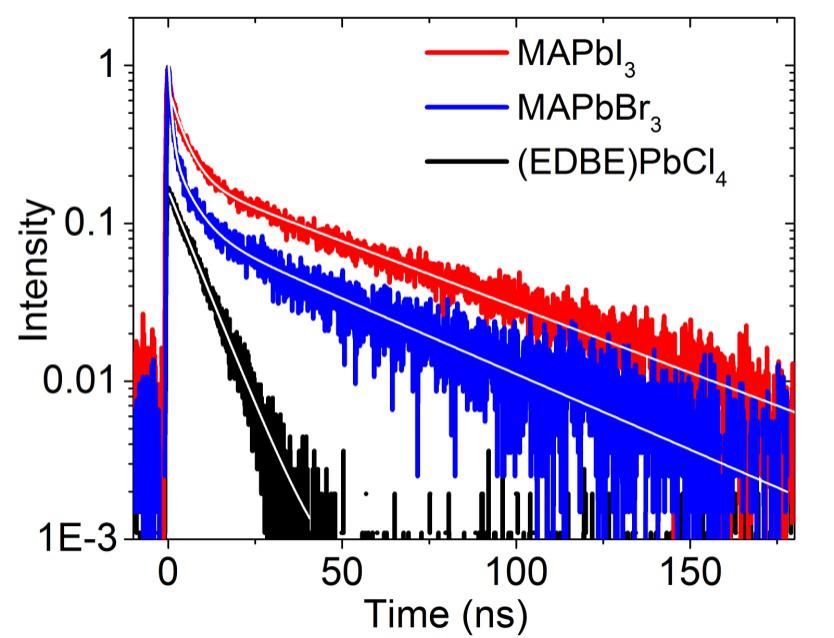


### Figure S6. Time resolved PL curves of MAPbI_3_, MAPbBr_3_ and (EDBE)PbCl_4_ single crystals. Excitation and emission wavelengths are reported in the text. All intensities were normalized, and the one of (EDBE)PbCl_4_ was further divided by a factor of five for clarity. The white lines in the curves are exponential fittings of the data.

### The decay curves of MAPbI_3_, MAPbBr_3_ and (EDBE)PbCl_4_ were fitted with double, triple, and single exponential fits, respectively. The resulting decay components of MAPbI_3_ are 4.3 and 52.2 ns, with contributions of 18 and 82 %, respectively. Those of MAPbBr_3_ are 0.8, 5.2, and 45.4 ns with contributions of 10, 18 and 72 %, respectively. While the longer decay times are consistent with the values previously reported for these 3D perovskites [4], our instrumental resolution (0.05 ns) allowed to resolve the additional presence of the fast components with decay times < 1 ns. Due to the limited time window, ultralong-lived components (> 300 ns) were not detected. Finally, (EDBE)PbCl_4_ decay has only one component of 7.9 ns, consistent with the photoluminescence lifetime reported for similar 2D perovskites [1]. Note that all the fast components are below 10 ns, much faster than those of commercial scintillators based on Ce^3+^ doped LuI_3_ [2] and LaBr_3_ [3].

### Pulse Height Spectra

Pulse height spectra were measured at room temperature (RT) under 662 keV gamma excitation from a ^137^Cs source (no. 30/2010, 210 kBq). The pulsed output signal from a Hamamatsu R2059 photomultiplier (PMT) was processed by a Canberra 2005 integrating preamplifier, a Canberra 2022 spectroscopy amplifier, and a multichannel analyzer. To improve the light collection efficiency the samples were coupled to the quartz window of the PMT with Viscasil grease and covered with several layers of Teflon tape. Light yield is obtained from the position of the 662 keV photopeak in pulse height spectra both recorded with the PMT and the APD. Using the PMT, the photoeletron yield, expressed in photoelectrons per MeV of absorbed γ-ray energy (phe/MeV), is determined by comparing of the peak position of the 662 keV photopeak to the position of the mean value of the single electron response [2].


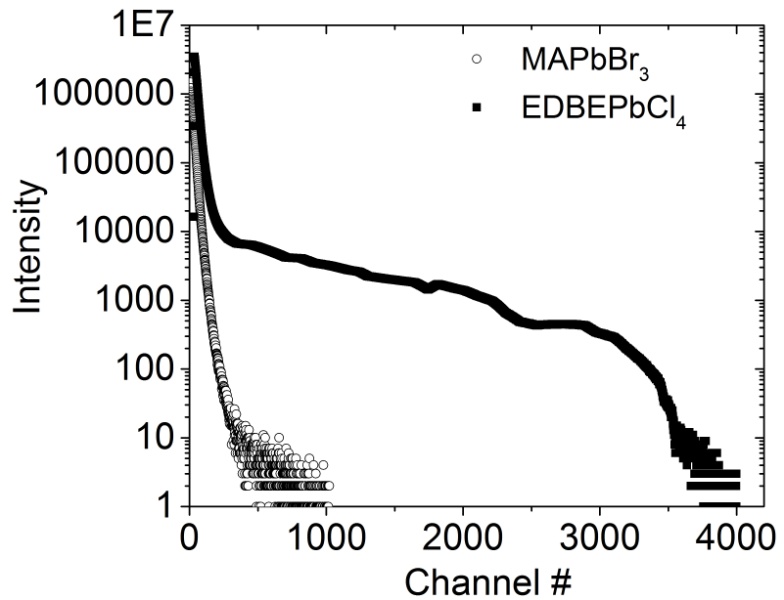


**Figure S7**. Pulse height spectra of perovskite crystals under 662 keV of ^137^Cs source with a pulse shaping time of 2 μs. The room temperature light yields derived for (EDBE)PbCl_4_ and MAPbBr_3_ are 9,000 and  < 1,000 photons/MeV, respectively.

# References

[1] Dohner, E. R., Jaﬀe, A., Bradshaw, L. R., and Karunadasa, H. I., Intrinsic white-light emission from layered hybrid perovskites, *J. Am. Chem. Soc.* **136**, 13154-13157 (2014).

[2] Birowosuto, M. D., Dorenbos, P., van Eijk, C. W. E., Krämer, K. W., and Güdel, H. U., High-light-output scintillator for photodiode readout: LuI_3_: Ce^3+^, *J. Appl. Phys.* **99**, 123520-1-123520-4 (2006).

[3] van Loef, E. V. D., Dorenbos, P., van Eijk, C. W. E., Krämer, K. and Güdel, H. U., High-energy-resolution scintillator: Ce^3+^ activated LaBr_3_, *Appl. Phys. Lett.* **79**, 1573-1-1573-3 (2001).

[4] Shi, D. et al., Low trap-state density and long carrier diffusion in organolead trihalide perovskite single crystals, *Science* **347**, 519 (2015).
